# A Qualitative Investigation of Parent Perceptions of Home Exercises for Congenital Muscular Torticollis

**DOI:** 10.3390/children11060689

**Published:** 2024-06-05

**Authors:** Audrey Stitt, Rebecca Operacz

**Affiliations:** 1 Physical Therapy Program, College of Public Health, Temple University, Philadelphia, PA 19122, USA; 2 Physical Therapy Program, Jefferson College of Rehabilitation Sciences, Thomas Jefferson University, Philadelphia, PA 19107, USA

**Keywords:** congenital muscular torticollis, physical therapy, home exercise program, parent perceptions, parent stress

## Abstract

The purpose of this qualitative study was to describe parent perceptions of the home exercise program (HEP) for infants with congenital muscular torticollis (CMT), and how these perceptions evolved over a physical therapy (PT) plan of care. Twelve participants were recruited from a pediatric PT clinic, and nine completed interviews at three time points. Qualitative description and an iterative approach for thematic analysis of 27 interviews yielded three themes that corresponded to the research questions. The participants’ responses were categorized into three main themes: (1) parents’ perceptions of three key exercises within the HEP, (2) internal and external sources of stress, and (3) sources of empowerment and disempowerment. Regarding the HEP, parents articulated common sentiments for three frequently prescribed exercises for the management of CMT: (1) tummy time was the fast favorite, (2) ipsilateral cervical rotation was perceived as stressful, and (3) contralateral cervical lateral flexion felt uncomfortable. Additionally, participants disclosed internal and external sources of stress (guilt, uncertainty, and the demands of returning to work) and sources of disempowerment (inconsistent messaging frompractitioners, feeling overwhelmed) and empowerment (being able to see the bigger picture and clear communication and education about the diagnosis) with respect to managing their infant’s CMT. These themes provide insight into the evolution of parent perceptions over a PT plan of care for CMT. Participants’ insights suggest a need for consistent messaging regarding the diagnosis and evidence-based management of CMT, addressing parent stress, and modifying how exercises are taught. This study contributes updated research on parents’ experiences with physical therapy and the HEP for their infant’s CMT.

## 1. Introduction

Congenital muscular torticollis (CMT) is a postural deformity present at birth, affecting 3.9–16.0% of infants [1,2]. It is characterized by unilateral shortening of the sternocleidomastoid muscle causing ipsilateral cervical lateral flexion and contralateral cervical rotation. Current evidence confirms that conservative management of CMT through physical therapy (PT) has excellent outcomes when initiated in early infancy [1,3,4], and that PT accelerates recovery [3]. Prompt identification and intervention is associated with a better outcome [1,3,4,5], lower healthcare utilization [5,6], a shorter episode of care [1], and prevention of secondary complications [4,7], which may positively affect long term results for infants with this condition.

Infants with CMT are at increased risk for physical deformity and delay across most developmental domains [4,8,9]. CMT may lead to craniofacial asymmetry, namely positional plagiocephaly, facial/ocular/mandibular asymmetry, and cervicospinal dysmorphism [4]. CMT and plagiocephaly have been estimated to coexist in upward of 75–95% of cases, and their effects are synergistic in perpetuating positional imbalance [7]. Infants with CMT and/or positional plagiocephaly have been documented to exhibit poorer movement repertoire [10] and gross motor delay as infants and toddlers [8,9,11]. These impairments have potential to carry over into school-ages [12]. Additionally, the presence of CMT and/or positional plagiocephaly in infancy has been connected to future cognitive and language delays [9] and attention-deficit/hyperactivity disorder (ADHD) in school-aged children [13].

Regarding emotional development, Hattangadi et al. found that healthy preschoolers whose parents experienced stress during their infancy had twice the odds of having a mental health condition by age three [14]. Parents of children with a health condition experience more stress than parents of healthy children [15]. Parental stress has been shown to influence childhood sleep, motor development, executive functioning, parent–child attachment styles, and self-regulation [16,17,18,19]. Increased parental stress is well documented in cases of chronic disease [15], but Oledzka et al. discovered that parents of infants with CMT experienced additional stress regarding their child’s diagnosis and treatment [20]. Additionally, evidence has shown that parents experience heightened stress when breastfeeding an infant with CMT due to positioning difficulties [20,21]. Importantly, parental stress and negative beliefs have an impact on adherence to medical and physical therapy management [22,23,24], which is especially crucial in the management of CMT.

To date, only Oledzka et al. and Rabino et al. have investigated the parental experience with, and adherence to, a physical therapy course of care for their infant with CMT [20,22]. Oledzka et al. highlighted parents’ experiences of stress and anxiety relating to the diagnosis and treatment for their infant with CMT, including performance of the home exercise program (HEP) [20]. Their study describes three unique challenges for the parent: correctly implementing the HEP, time management related to the HEP, and difficulty breastfeeding [20]. Rabino et al. found that a parent’s perception of the threat of their infant’s CMT increased the likelihood that they remained adherent to performing the HEP and attending PT sessions [22]. Interestingly, they also found that parent involvement style (autonomous v. passive) influenced participation and follow up with the HEP [22]. While both Oledzka et al. and Rabino et al. identified important factors influencing the parent experience while managing their infant’s CMT, neither study investigated parents’ perceptions about the specific evidence-based interventions assigned to them in their HEP.

The clinical practice guideline (CPG) released by the American Physical Therapy Association provided a comprehensive standard of care for the physical therapist [1]. It included five “first choice” interventions: passive cervical range of motion (ROM), active cervical and trunk ROM, facilitation of symmetrical movement, environmental adaptations, and parent education [1]. Passive cervical ROM, or “manual stretching”, was the most common intervention when addressing CMT and had excellent outcomes when performed at high frequency and supplemented with active ROM exercise like prone play (“tummy time”) [1]. It is impossible for the PT to meet the recommended high frequency in isolated weekly or biweekly visits. Therefore, parental adherence, as defined in this study as compliance with the HEP, is crucial in achieving timely and optimal outcomes.

The current literature clearly indicates that CMT is linked to a child’s motor, cognitive, emotional, and physical development, and that downstream effects of CMT can influence development across the lifespan. Given this information, the purpose of this study was to understand parents’ perceptions of the three most common exercises for CMT and to identify factors that influence parent adherence to the home exercise program. A secondary purpose was to understand how parental stress may influence the parent and their infant throughout the course of care. Our research questions were:

1. What are parents’ perceptions of the three most common evidence-based home exercises?
2. What are parents’ perceived stressors regarding their infant’s diagnosis and home management of CMT?
3. How do parents’ perceptions and stress regarding CMT and home management for CMT evolve within the episode of physical therapy care?

## 2. Materials and Methods

### 2.1. Design

Qualitative interviews with parents of infants with a diagnosis of CMT provided the means of answering the research questions. This study employed qualitative description to understand parents’ perceptions and stress related to the HEP for infants with CMT [25]. This methodology allows exploration of phenomena with limited evidence or understanding, and it allows “researchers to stay close to the data” through continuous analysis [25] (p. 2). Semi-structured interviews were conducted using a template (see Supplementary Materials) to elicit a rich description of participants’ perspectives on specific home exercises for their infant with CMT and to identify the perceived sources of stress across three consecutive months within each participants’ plan of care. The interview template provided a consistent approach to every participant’s interview, but probes differed based on participants’ responses over the three points in time. Sample probes asked about parents’ confidence handling their baby and about help or support available at home.

### 2.2. Sampling and Participants

Participant recruitment and data collection occurred on a rolling basis, dependent on clinic referrals from March 2022 to January 2023. Twelve participants were recruited from a pediatric physical therapy (PT) clinic in a mid-Atlantic urban area. This clinic was chosen based on the demographics of its patient population, which was representative of the surrounding community, and the clinic’s established clinical teaching partnership with the primary author’s institution. While parent demographics were not collected, the participants’ rich interview data indicated a broad representation of the experiences of parents pursuing care at this pediatric clinic. Participants with a diagnosis of CMT attended the clinic per a physician referral. During their first visit, they were provided an informational handout describing the study. Interested participants left their name and phone number and the primary researcher (AS) contacted them to complete formal study consent. See Table 1 for participant and infant characteristics, including the severity classification for CMT as defined by the CPG [1].

### 2.3. Inclusion/Exclusion Criteria

Participants were included in this study if they had the ability to regularly attend PT over the course of three months, had an infant with a diagnosis of CMT, and met criteria for “early” grade CMT. Additionally, each participant had to be able to meet by phone or virtually to complete three interviews of 15–30 min each month of their individual plan of care. Exclusion criteria were infants who did not meet the criteria for “early” grade CMT [1].

### 2.4. Procedure and Data Collection

Following referral to physical therapy and evaluation by a physical therapist, participants engaged in an individualized plan of care. Participants were treated by one of two physical therapists, one with 12 years of experience and one with two years of experience plus additional continuing education specific to pediatric rehabilitation. Both PTs employed best practice standards of care for the treatment of CMT, as outlined in the CPG [1] and balanced by individualized treatment approaches dependent on the needs of each family. One of two physical therapists instructed parents in the appropriate performance of the home exercises (tummy time, ipsilateral cervical rotation, and contralateral cervical lateral flexion) and provided either written or video instructions for the parents to complete the exercises daily at home. Weekly physical therapy sessions were weaned to every other week and then monthly, pending the infant’s progress and the parents’ confidence with the HEP. 

The researchers conducted semi-structured phone interviews with participants at three time points during their PT episode of care. Interviews were audio recorded and transcribed via the Microsoft Word^®^ transcription feature (version 16.69). Transcriptions were cleaned for fillers, such as “like”, for clarity. Initial interviews were scheduled within one week from PT initial evaluation, and at one-month intervals thereafter for a total of three interviews. Interview questions focused on how parents perceived the three prescribed exercises. Additional questions prompted participants to share their thoughts and experiences with CMT, stress, family dynamics, feeding difficulties, the referral process, and other concepts that emerged across interviews.

**Figure 1:**
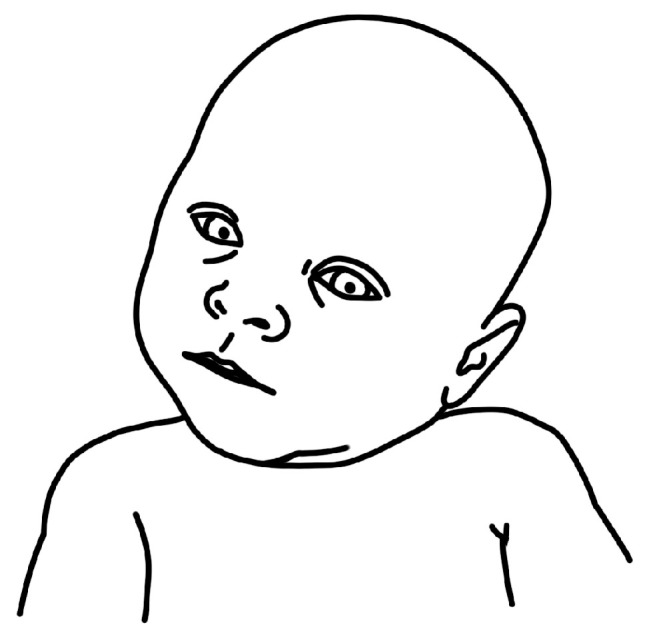
Schematic depiction of left-sided torticollis.

**Figure 2:**
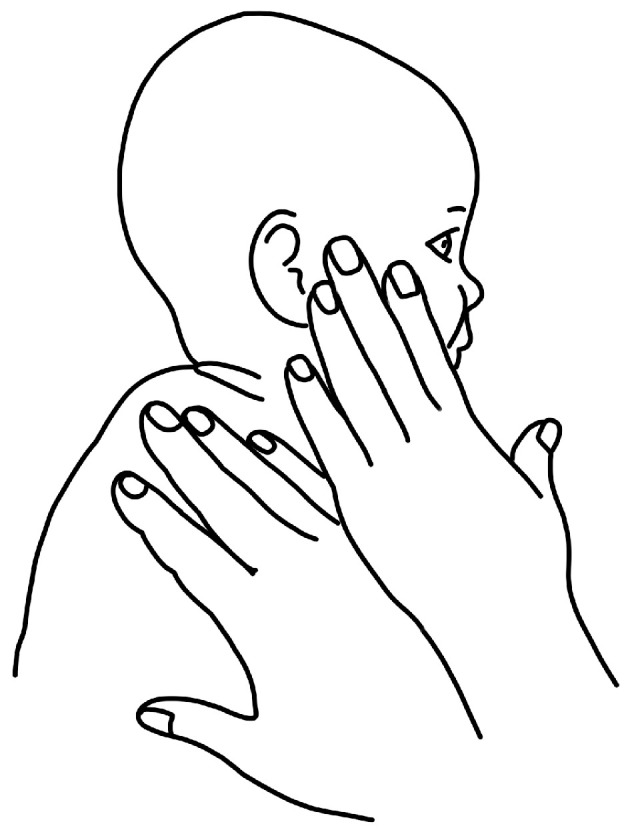
Schematic depiction of left ipsilateral rotation manual stretch.

**Figure 3:**
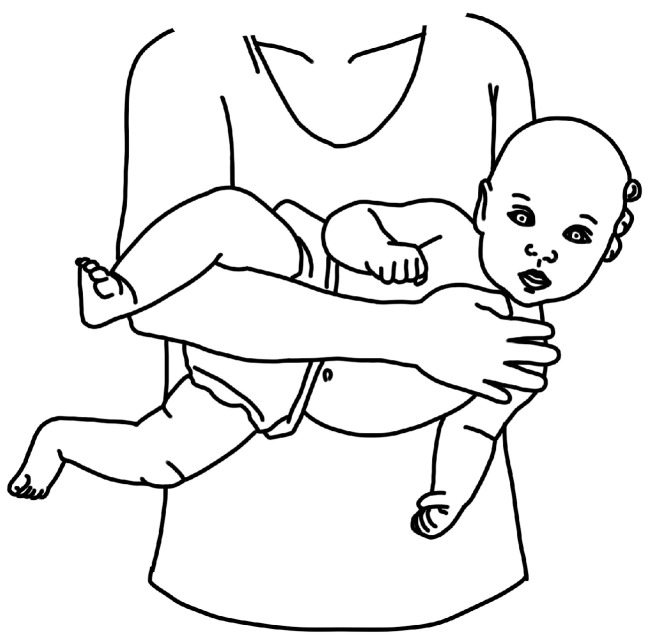
Schematic depiction of “football hold” for contralateral cervical lateral flexion.

**Figure 4:**
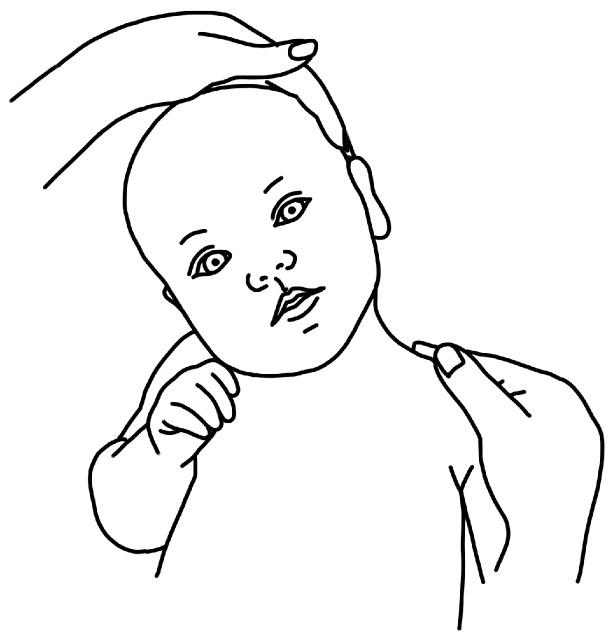
Schematic depiction of supine contralateral cervical lateral flexion manual stretch.

Figure 1, Figure 2, Figure 3 and Figure 4 depict left-sided CMT and three examples of stretching exercises that were prescribed as part of the plan of care for each participant.

### 2.5. Data Analysis and Thematic Development

Data in the form of three audio-recorded, transcribed interviews included infant demographic data and parents’ responses to questions. CMT severity scores were provided by the participants’ PT. Data were stored in a password-protected electronic folder on the primary author’s (AS) institutional server. Interviews were conducted by the primary researchers (AS and RO). One of the authors, RO, has experience and training in qualitative data collection. An outside academic consultant with qualitative research expertise also assisted with development of the interview template and trained the primary author, AS, in qualitative interviewing. Researchers AS and RO individually read and re-read all 27 transcripts, representing nine participants completing three interviews each. The researchers maintained a codebook, and data analysis occurred during each interview via memos, during each researcher’s individual review of transcripts, and in monthly research group meetings. Using qualitative description [25], each researcher engaged in a multi-cycle, iterative coding process for each transcript, yielding the categories and themes as presented in our results. Monthly research meetings were conducted to review transcripts as a team and discuss coding discrepancies to achieve inter-coder agreement. We identified key themes, representative of both the rich meaning of the participants’ words as well as the phenomenon in question: parent perceptions of the HEP. Member checking, a means of qualitative validation [26], was completed by five out of nine participants. All participants received an email invitation to provide written comment on the final themes, and five participants responded with their feedback. Through member checking, we sought to ensure accurate representation of the participants’ perspectives with our final themes. All member checkers confirmed that the final themes accurately represented their thoughts and experiences.

Approval for this study was granted by the Internal Review Board of the primary author’s institution (approval number 29291). The informed consent provided to participants is available upon request from the authors.

## 3. Results

Out of the twelve families identified by the clinic owner as appropriate for inclusion in this study, three failed to respond to follow-up by the primary researcher, yielding nine participants who completed the study. We collected basic background information regarding infant characteristics. This information confirmed that we had adequate representation of severity and infant characteristics commonly associated with CMT diagnosis, with the exception that we were lacking participants with a severity classification of Early Severe (Grade 3) [1]. The clinic had not received any referrals with a diagnosis of “Early Severe” within the study time frame. Three parents commented on a difficult labor, eight infants were firstborn, four were female, and only one parent reported significant spit-up (or reflux). Five infants had a severity classification of Early Mild (Grade 1), and the other four were classified as Early Moderate (Grade 2) [1] (see Table 1). 

Thematic analysis yielded three primary themes regarding parents’ perceptions of the PT plan of care for their infant with CMT. These themes were related to perceptions of the three primary exercises described in the CPG [1], parents’ sources of stress, and parents’ sources of empowerment and disempowerment in managing their infants’ diagnosis.

### 3.1. Perceptions of the Three Primary Exercises

Parents consistently indicated a familiarity with tummy time prior to the formal recommendation as part of the CMT plan of care. Although stressful at first, it quickly evolved into a “favorite” activity that endured throughout the episode of care. While all participants expressed that it was initially hard to watch their babies struggle, using positioning aids and toys allowed it to become more “intuitive” and a regular part of the infant’s routine. All participants found that success in tummy time was visual “proof” that their baby was gaining strength, and it became a method of play that was both enjoyable and therapeutic by the third interview time point.

Ipsilateral cervical rotation was stressful for parents due to the baby’s distressed reaction during the stretch and the need for hand placement on the baby’s face. Parents disliked “forcing” their babies into rotation, but over time, parents found strategies to alter this exercise by using visual tracking with stabilization, which was less stressful than manually stabilizing the baby in supine. The CPG clearly states that the infant should not be crying or resisting a stretch [1]. The mismatch between the PT skillfully performing the stretch and the parent attempting it at home contributed to parents’ perceptions that they were not doing it correctly. A consistent thread across participants during the first interview was that it was unnerving to do something “to” the infant. The second time point illustrated a shift in completing this exercise with modifications, albeit less frequently due to continued parental hesitancy with the exercise and parents’ reprioritization of exercises toward active cervical and trunk ROM interventions.

Parents consistently reported limited adherence to contralateral cervical lateral flexion. Visualizing tense neck muscles and observing the baby’s discomfort caused parents to doubt that they were doing the exercise correctly, and thus several participants stopped this stretch once their babies resisted or cried. Overall, participants reported that this stretch felt counterintuitive during the postnatal time during which they were trying to heal from the birth process, attune to their babies, and bond as a family. They felt guilty doing this exercise and then guilty for not doing it. Words that described contralateral cervical lateral flexion included “wailing baby”, “angry baby”, or “fear of dropping baby” (when performed as a football hold, see Figure 3). Table 2 illustrates the evolution of parent perceptions of the HEP. 

### 3.2. Sources of Stress

Participants identified stressors that formed themes of internal vs. external sources of stress. All the exercises were difficult and stressful for infants and families in the first month, especially the passive ROM exercises that required the parent to handle the baby’s head. Parents’ fatigue, difficulty breastfeeding, making sense of the diagnosis, and managing appointments all added to the experience of stress. These factors contributed to the emotionality of obtaining a diagnosis of CMT, wanting to protect their infant, and wanting to “fix” them, all at the same time. Table 3 illustrates the evolution of stress across the episode of care. Specifically, internal stressors arose from the parent feeling responsible for their infant’s diagnosis of CMT, worry over not doing home exercises correctly, and guilt for not doing cervical flexion/rotation manual stretches with their baby. External stressors included return to work, information on the internet, extended family input, and scheduling demands. Several participants shared that they sought out information on the internet and were afraid of the images that they saw. 

In contrast to these external stressors, one participant (P7) commented during the second interview “I just think there’s a lot worse things to deal with that could happen with a newborn…. I’m just thankful”. This response supported this participants’ interpretation of stress as an expected part of parenting a newborn and not specific to the diagnosis of CMT. Interestingly, Bassi et al. (2021) differentiate in their study between “general” parental stress, such as finances, marriage, and parenting, from parenting stress relating to the management of a child’s health condition [23]. Participant 7’s perspective (minimizing concern specific to their baby’s CMT diagnosis in the context of other parental stressors) contrasted with the other participants who identified internal and external stressors specific to their infant’s CMT diagnosis.

### 3.3. Sources of Empowerment and Disempowerment

Coupled with the impact of stress from various sources, participants’ perspectives indicated sources of empowerment or disempowerment specific to managing their infant’s CMT, which are illustrated in Table 4. During the first interview, parents expressed relief in meeting the PT who provided education, established a clear plan, and taught exercises that were individualized and modifiable, including the use of video recording for ideal teaching and learning. The therapeutic alliance promoted trust. During the second interview, parents expressed a sense of pride and accomplishment in completing the HEP, even when they reprioritized specific exercises to suit their infants’ and their own preferences.

Soon after diagnosis, many parents were concerned with the potential for developmental delays but struggled to understand how stretching (ipsilateral cervical rotation and contralateral cervical lateral flexion) would help their baby. By the second time point, many parents shared relief in observing progress and pride with attaining developmental milestones. During the third interview, all parents expressed an understanding for the connection between the HEP and positive developmental outcomes, which was not evident in the first two interviews. This ability to “see the bigger picture” empowered parents to feel responsible for the positive change observed in their infant’s CMT diagnosis and in their overall gross motor development.

Disempowering factors stemmed mostly from misinformation or inconsistent information about CMT. Conflicting recommendations from various health professionals limited parents’ sense of control over their baby’s outcome, created more uncertainty and stress, and minimized parental input into the clinical decision-making process. Participants reported confusing information from other healthcare providers who recommended doing the exercises independently at home, taking a “wait and see” approach, or performing alternative exercises of body work and manual techniques. Parents had a strong desire to “fix” things, but delays in referral and conflicting information left parents feeling hindered in their efforts to address the CMT. 

Our study’s participants also expressed frustration with delayed referral and perceived that they were not being listened to by their provider. Additionally, parents received ambiguous information regarding their baby’s need for a cranial orthosis (“helmet therapy”), which is a conservative approach to correcting craniofacial dysmorphism, including positional plagiocephaly. They felt disempowered by the uncertainty of managing their infant’s craniofacial dysmorphism, scheduling appointments, the quantity of exercises and stretching techniques, and some providers not prescribing any exercises at all. 

An additional disempowering factor involved distress related to the HEP and returning to work, which felt like “a juggling act” to participants. Parents felt obligated to perform exercises or were constantly thinking about their infant’s positioning. Parents whose infants were diagnosed later discussed feelings of frustration for the delay in detection and getting started on the HEP. They reported less time for the HEP in an overwhelming return-to-work schedule. Overall, as parents returned to work, their stress shifted away from the HEP and more toward family dynamics. By the final interview, parents only completed tummy time and activities related to milestone achievement. A sense of confidence and self/family advocacy developed consistently across all participants by the final interview so that feelings of disempowerment decreased over interview time points.

## 4. Discussion

This is the first study to describe how parents perceive specific CMT exercises across an episode of care. It supports the work of Oledzka et al. and Rabino et al. [20,22] and offers insight into parents’ perceptions and stress across the PT episode of care for their infants with CMT. Participants offered insights into the common exercises prescribed as part of a home exercise program for CMT, and these perceptions shed light on parents’ adherence or hesitance to perform specific exercises with their infants. Additionally, we identified sources of parental stress and factors that either hindered or facilitated parents’ adherence to the HEP. Empowering factors that fostered confidence in managing the CMT diagnosis included early participation in PT and being able to see the bigger picture. Conversely, disempowering factors (e.g., frustration over conflicting information from various health care providers) served as a barrier to HEP adherence. 

The time frame for interviews was chosen to capture potential changes in parents’ perceptions during their respective episodes of care for their infants with CMT. Early on, several participants described barriers to completing the HEP, such as wanting to accommodate their infant’s irritability, not knowing how far to push with the stretches, and fear of performing the stretches incorrectly. Parental perceptions at interview time point one included a sense of guilt, coping with a long, traumatic labor, and processing news of the CMT diagnosis. In month two, babies demonstrated improvement, so parents tended to stop the intensity of the HEP, which is consistent with other studies [20,27]. Once babies entered PT and demonstrated growth and progress, parental anxieties diminished, although participants communicated ongoing hesitancy and uncertainty with performing manual stretches. Parents lacked confidence with handling techniques for manual exercises out of fear of doing “to” rather than doing “with” their baby. Inconsistency in performing the two cervical stretches contrasted with tummy time, which parents reported felt more natural and more easily incorporated into play. Tummy time promoted engagement and attunement with their baby [28], thus potentially contributing to increased frequency of this home exercise.

While participants had the freedom to navigate parental roles at time point one (guided by internal stressors), time constraints by time point three caused parents’ priorities to shift toward juggling the work, life, and parenting balance (external stressors). Internal stressors may have influenced parents to adhere to exercises more consistently, as was noted at time point one. In contrast, at time point three, external stressors may have detracted from overall exercise adherence. These stressors relate to the themes of empowerment and disempowerment described by the participants across all three time points.

Factors that contributed to parental empowerment included early referral to PT, a therapeutic alliance with their PT, seeing the bigger picture, and observing milestone achievements. Participants expressed that a concrete plan and clear communication enhanced their adherence and confidence to perform the HEP for their infants. Conversely, factors that contributed to parental disempowerment included conflicting or ambiguous information from various sources, feeling overwhelmed, worry over not doing enough, and challenges with managing time. 

Kaplan et al. and Stellwagen et al. affirmed that early identification and referral to a PT by the pediatrician is critical for CMT resolution [1,2]. However, our study confirms findings by Oledzka et al. that there is often a delay between diagnosis and initial PT evaluation [20]. Over half of the participants in the Oledzka et al. study were advised to wait until the infant’s next pediatrician visit, and many were directed to administer interventions at home with reference to YouTube™ videos [20]. Additionally, we found that three parents pursued non-evidence-based interventions to address their infant’s CMT. Healthcare providers who use different approaches including manual therapies and bodywork should conduct high quality research to confirm or deny the use of these approaches for CMT treatment prior to using them clinically [27].

The recently published CPG indicates that stretching should be stopped if the infant resists [1]. Training parents in the HEP can include more detail on the degree and intensity of stretching, stretching in a graded fashion, and teaching parents what constitutes an acceptable physiological response. Palmer and colleagues assessed parent efficacy for using prone positioning for their infants [28]. They found that parents who used preparatory touch and infant attunement demonstrated increased adherence to tummy time since the infant was more accepting of it [28]. Parents who were taught a stepwise approach to prone positioning, in which the infant had the opportunity to demonstrate acceptance of each step prior to moving on to the next, increased the frequency of tummy time [28]. These subtleties can be addressed in training resources, including telehealth, which can decrease parental stress and increase adherence.

The contrast between Participant 7’s lack of diagnosis-related concern and other participants who catastrophized the diagnosis based on internet searching may indicate a need for more consistent and accurate information for families. Our participants’ voices indicated a need for evidence-based resources to manage the stress associated with a diagnosis of CMT. Unlike chronic conditions, acute conditions such as CMT require a time-sensitive approach that may require more emotional support in the short term. The PT is in a unique position to discuss the stressors associated with the postpartum period, help to minimize guilt, and assist in empowering parents in the resolution of CMT.

### 4.1. Implications for Practice

While concrete or generalized recommendations cannot be provided from this data, our participants’ perspectives raise important considerations regarding the HEP for CMT. (1) Tummy time may be more accessible for parents because it promotes infant bonding and attunement through play, especially after the first month. However, it is anxiety provoking due to the infant’s poor head control, worry over the infant’s breathing in this position, and related concern for sudden infant death syndrome. Teaching parents the important connection between tummy time and the cascade of benefits associated with it is critical in the first visit [29]. (2) Ipsilateral cervical rotation may be better received by the parent if it incorporates education about gentle pressure, visual gaze, and brief holds in the first month. Once visual tracking is more mature, sustained pressure can be offered to enhance cervical rotation. (3) Contralateral cervical lateral flexion is stressful for the parent, which may significantly reduce parental adherence. Ensuring that parents demonstrate confidence prior to leaving the teaching session and having individualized modifications for positioning may decrease parent stress and improve adherence to this exercise. 

### 4.2. Limitations

This study has several limitations. First, the sample was drawn from one clinic in an urban region, and the participants’ voices may be limited to the perspective of individuals within a metropolitan setting with a high density of healthcare resources. Potential barriers to care such as transportation and proximity to providers were not discussed during these interviews and may pose more of a concern for families in suburban or rural communities. Additionally, because participants and providers were not blinded to each other, it is possible that the therapeutic relationship was altered. For example, there is potential bias for the provider to have adjusted treatment for families involved in this study, or for the participants to be more vocal about treatment after their interviews.

As this was a qualitative descriptive study exploring parental perceptions of the HEP for CMT, we cannot generalize findings beyond the experiences of these nine participants. Although these participants were recruited from a clinic that serves a culturally and ethnically diverse region of a major urban area, participant demographics were not explicitly collected. Without this data, we cannot extrapolate the impact of demographics on the healthcare experience for families coping with a CMT diagnosis. 

While perceived parental stress may not be specific to the diagnosis of CMT for all participants, longitudinal tracking across interviews in this study provides useful insight into the role that stress plays throughout the first few months of new parenthood. While we aimed for a broad representation of family experiences with CMT, we were only able to recruit participants with Early Grades 1–2 CMT [1]. Future work should explore the impact of higher severity CMT on parents and families. Finally, the original intent of this study was to determine more information about the exercises, such as the extent of sustained holds, repetitions, and frequency, but participants did not keep track of these details and reported dosing and intensity in more functional terms.

## 5. Conclusions

Overall, this study has added to the body of pediatric rehabilitation literature. First, this study provides insight into how parents navigate the most common, evidence-based exercises for CMT over the course of a PT plan of care by investigating how parental perceptions of each exercise influence adherence to the HEP. Second, the longitudinal nature of our study demonstrated how sources of parental stress change across the early months of infancy, which impacts the availability of parents to attend to their baby’s PT plan of care. Third, our participants provided insight on the ways that parents are empowered or disempowered by the current beliefs and practices of families and the health care system specific to CMT. Fourth and finally, this study highlights how consistent and deliberate CMT education is needed to foster trust between the health care community and patients and families. Pediatric PTs are still in the early phases of training community providers, offering parent support, and addressing the subtle yet significant elements of the HEP in CMT.

## Figures and Tables

**Table 1:** Participant characteristics.

Parent Characteristics									
Participant	P1	P2	P3	P4	P5	P6	P7	P8	P9
Single or couple interview	Single *	Single	Single	Single	Single	Single	Single	Single	Single
1st child?	Yes	Yes	Yes	Yes	Yes	No	Yes	Yes	Yes
Comment on labor and delivery	Yes	No	No	Yes	No	No	No	Yes	No
Concern for reflux	No	No	No	No	No	Yes	No	No	No
Infant Characteristics									
Participant	P1	P2	P3	P4	P5	P6	P7	P8	P9
Gender	Male	Male	Female	Male	Female	Female	Female	Male	Male
Age at diagnosis in weeks	4	6	4	3	10	4	11	12	16
Age at 1st PT session (weeks)	7	9	6	3	10	6	11	12	21
Severity classification	Mild	Mild	Mild	Mod.	Mild	Mod.	Mod.	Mild	Mod.

* All 9 participants were interviewed individually, with the exception (*) that P1’s partner joined for the second interview.

**Table 2:** Evolution of parent perceptions of the HEP.

**Theme 1: Tummy Time: The Fast Favorite**		
Exemplar data support: I feel like tummy time has become … we’re just playing with her as a baby. I think the other two stretches really feel… different than play time. (P1)		
Time point	Additional participant quotes	Codes
T1	Tummy time wasn’t very happy time initially … but over time, she has become stronger. (P6)	Familiar activity Better over time
T2	Tummy time is going significantly better since we started the PT. In the beginning he literally hated it, and now I have so many pictures of him on his belly. (P8)	Intuitive Success with tummy time gives hope
T3	When we play, we do tummy time. (P1)	Play time Natural
**Theme 2: Ipsilateral Cervical Rotation: More Stress Than Play**		
Exemplar data support: It’s easier to get her to do it in different ways; you don’t have to just hold her head. She could follow a toy or follow my face, so you can do it while playing versus having to *make* her do it (P3)		
Time point	Additional participant quotes	Codes
T1	Well, my baby gets really angry when I turn her head…she doesn’t care for stretching, she just gets annoyed. (P5)	Stressful Baby hates it Possible to incorporate into play
T2	It definitely feels less forceful and more motivating. I think being able to make eye contact or use the toy feels like a big difference. With a three-week old you can’t encourage them, it’s only your hand. (P4)	Still forced Using strategies (i.e., toys) to accomplish motion
T3	I’d rather do the stretching her arms overhead, those feel a little more functional, a little more movement going on. (P1)	Low priority Progress with head turns = less urgency to do exercise
**Theme 3: Contralateral Cervical Lateral Flexion: Deliberate and Uncomfortable**		
Exemplar data support: …it’s the only exercise that feels like “PT” …I guess it’s not always the one we rushed to do. (P1)		
Time point	Additional participant quotes	Codes
T1	Ear to shoulder is definitely more awkward to do because you have to do it holding her up. It’s more intentional… I mean you can get her to turn her head with a toy, but you can’t get her to put her ear to her shoulder with a toy. (P3)	Awkward Intentional
T2	It’s not my favorite, and she doesn’t like it, so that doesn’t make it any better. (P5)	Not becoming play Not natural
T3	Ear to shoulder is just not a natural movement like when she’s playing. (P3)	Not performed

**Table 3:** Evolution of parental stress related to CMT.

**Theme 1: Guilt and Uncertainty as Internal Sources of Stress**		
Exemplar data support: I think just the overall stressor is that we feel like we just want to enjoy him being a baby, and we’re constantly like, ‘oh, he’s lying on his back, we should pick him up or we should do the exercise’. If we have any down time, I know we both feel like we can’t just play with him because we’re constantly thinking about, ‘well what *should* he be doing?’ (P2)		
Time point	Additional participant quotes	Codes
T1	…birth is something that my body is supposed to know how to do, but I wasn’t able to do, and in not being able to ‘do’, I hurt my baby. (P8) So we went home and tried to do it a few times after that appointment, and she wasn’t as upset or anything so we thought we weren’t doing it right and we were worried… are we hurting her by not making her cry? (P1)	Guilt Blame Uncertainty
T2	He loves to lay on his back and kick around. He loves it so much, but every time he’s doing it, I’m like, ‘I need to pick him up and re-position to be on his back’ or ‘he’s looking the wrong way’. (P2) I don’t do it as… regularly as I should… I know it’s only hurting [baby], and I feel terrible about that. But I’m also like ‘I can only do what I can do’. (P9)	Guilt for not doing enough Diagnosis anxiety
T3	Like ‘oh am I doing this right? Is this the way it’s supposed to go?’ (P5) Sometimes we’re scared to advocate for ourselves, but I cannot be scared to advocate for him. (P8)	Uncertainty Advocacy for baby Recognition of need for early mitigation of guilt
**Theme 2: Work, Family, and Google as External Sources of Stress**		
Exemplar data support: … we’re flying by the seat of our pants trying to figure out what will work best. (P5)		
Time point	Additional participant quotes	Codes
T1	“We Googled it—big mistake … the images on Google are really scary. No, Googling didn’t make me feel better. Googling freaked me out”. (P2)	Overwhelmed
T2	Between trying to introduce solids and the bedtime routine I’m just like, “oh we have to do these stretches”, and I try to squeeze them in and they’re just a little bit more of an afterthought. (P9) My [partner] actually lost a family member to SIDS [sudden infant death syndrome]… So going into anything about repositioning and flipping and turning and pillows and anything felt very scary (P8)	Overwhelming Fear of past trauma/SIDS Time commitment Return to work
T3	… when we were both sleep deprived, I think that was probably the toughest part, but now that we’re getting a little more sleep, it’s starting to get a little easier. (P7) But the past two weeks especially I’ve just been like ‘there’s not enough time, we gotta do this bedtime routine, and we got to get you to sleep’ (P9)	Work as a barrier Lack of sleep Need for flexibility

**Table 4:** Sources of parental empowerment and disempowerment.

**Theme 1: Sources of Parental Empowerment**		
Category 1: PT = Relief		
Exemplar data support: I think it was a big leap after PT because we had a plan, and we got the proper care for her… So I think that the PT visit was definitely a big ‘OK, we can do this’. (P1)		
Time point	Additional participant quotes	Codes
T1	…you watch and if it doesn’t feel right this way, you can do it that way to modify… all sorts of information that we never got with the pediatrician. With the PT we don’t feel any unease about how this works. (P5) …for me at least, having those [instructions] “these are the three to five things that you can do for this amount of time this many times a day”, having that concrete instruction alleviates some of the stress for me. (P9)	PT education PT plan = reassurance Positive therapeutic alliance
T2	… and he has loosened up so much it really has helped even in the two weeks that we’ve been in PT it’s just been helpful to identify something that we can do that feels like it’s actually helping and being able to see immediate improvement. (P8) It’s kind of nice to have someone who knows some of the things that you don’t, and not to be paranoia-ing-ly Googling things all the time…which is just a bad idea…it’s nice to have a resource. (P3)	Belief in PT PT as an information filter
T3	I knew what to expect in PT sessions which was so nicely consistent and then as we watched [baby] master certain things we moved on to ‘oh this week you’re going to work on this’, so I knew we were getting better. (P5)	Being listened to Consistent information Matter-of-fact guidance Confidence to scale back from PT
Category 2: Seeing the Bigger Picture		
Exemplar data support: Seeing her rolling really shows you how important it is for her to be able to look in both directions. So that was like “oh I’m not just doing this to stretch your neck out”. (P3)		
Time point	Additional participant quotes	Codes
T1	…And flat spots … I hate to say it but there’s a cosmetic aspect that motivates me. … the other thing that is highly motivating is that [the PT] mentioned that sometimes these children can fall back developmentally so I became really committed to the PT regimen. (P5)	Seeing a difference Increasing confidence
T2	In dealing with this, we know that she will recover, and she will be OK. (P7)	Advantageous for general development Normalizing CMT difficulties with parent support groups
T3	Well, she started to be able to sit…And when she mastered that, I felt really good about it but perhaps most importantly, when she started to reach for toys at her midline I thought, ‘OK we’re good’. (P5)	“It’s curable” Hitting milestones Baby = strong
**Theme 2: Sources of Parental Disempowerment**		
Category 1: Medical Community Chaos		
Exemplar data support: Before I met with the PT and really understood how to do the exercises—what I should be doing, how I should be doing them, and for how long and how often—I just was like OK we’re supposed to be doing these stretches, but I didn’t really know what that meant … so that lack of understanding made it harder for me to remember to actually do it. (P9)		
Time point	Additional participant quotes	Codes
T1	[The pediatrician] was showing the exercise …. It was very stressful. She was very … heavy-handed, I mean, I know that babies aren’t fragile, they are getting poked and prodded and everything, but it’s her *neck*. So, we felt kind of unsettled, and she started to cry and turn bright red and I wanted to know: if you want her neck to be strengthened, we have to do *this*? (P1) For us, I feel like the physician said one thing about how long it should take, and the PT said another, and the [other provider] said something else. (P4) [From the pediatrician], we didn’t get a lot of instruction of how to do the exercises. At the time I thought I knew what I was doing and then when I went to the PT visit and worked through the exercises and the stretches with [the PT], I definitely was like “Oh, I was not doing this effectively”. (P9)	Delayed referral Physician prescribed exercise Not feeling heard Conflicting or ambiguous information (from providers, internet, and family)
T2	Having the appointment felt stressful because we weren’t sure what was going to happen, and finding providers… Do you go to PT… the chiropractor … myofascial? And you’ve been given so many suggestions, so sorting through all of that while trying to adjust to everything else is really stressful. (P4) They [PT and another provider] both said they were trying to reach the same goal, but one was a stretch with the muscle and the other was a stretch against… so we were like it doesn’t make sense to do both because how will we ever know if we are keeping it from progressing by doing both…. (P4)	Lack of shared decision making Non-EBP practices Negativity from other providers
T3	We just weren’t meshing well … I feel like she [healthcare provider] was using scare-tactics. I was feeling very overwhelmed every time we left because it was like there were a million things wrong with my baby. (P2)	Lack of alliance with healthcare provider (non-PT)
Category 2: Feeling Overwhelmed		
Exemplar data support: “when the PT gave us handouts of the exercises, it was showing a lot of different ways to do it, but I did get overwhelmed looking at all the images, like I would have just rather seen three different things to do without all the options. (P2)		
Time point	Additional participant quotes	Codes
T1	Without PT we would not have been able to learn about his specific type of flat head, and so we would have been overwhelmed and internalizing a lot of guilt…. (P8) So then trying to think about an additional appointment to another provider, and then sift through that information felt overwhelming. It was like, do we have to do this? Should we do it now? You know, when your baby is like 2 and ½ weeks old. No one could tell us for sure. (P4)	Overwhelmed Frequency and variations of exercises Finding pertinent information about diagnoses: CMT/PP
T2	I think my stress is not so much about not knowing what’s going on with her now, but more making sure I’m doing enough with her all day. When you do PT I think it makes you a little more aware of OK am I putting her down too much? Is she in this chair too long? … So I’m just stressed about what to do all day every day. (P2) But the flatness of the head and knowing that there is the potential for cognitive issues was really stressing me out because … I don’t know what I can do about this other than just get him the helmet…. (P9)	Am I doing enough Resignation to do uncomfortable exercise Increased number of exercises Concern for cranial molding peaking
T3	… he also had brachycephaly, which was a direct result of his positioning in my womb. So, we have been working on the back of his head but also the side of his head, which is overwhelming, …but being able to talk through the trauma of my birth with the PT and him being stuck really helped…. (P8)	Work schedule vs. child’s schedule
Category 3: Challenges of Time Management		
Exemplar data support: … so early detection is useful. Even if we figured this out at, let’s say, six months age, by that time both of us would be working again full-time…so if you can catch something early on that is probably better. (P6)		
Time point	Additional participant quotes	Codes
T1	When I am at home we generally split up … I basically give a few hours in the morning, some hours in the evening. … if I’m going to the office then [spouse] plays with her and she does activities with her at home. (P6) … between all the things in our life, all the different stuff, it’s been harder to find the right time to do it. (P4)	Division of labor (parent dyad) Managing new life routine
T2	Especially since the first month, when we only had 3 exercises to do and now we have a lot more. It’s a full-time job. The time commitment is incredible, and then there’s more stress because of the added things to do. (P2)	Sleep routine Return to work Juggling act
T3	I don’t feel like the PT part was stressful. It’s more time management and figuring out schedules and getting sleep. (P7) I’m sure if I was working, or if my partner—he’s very busy at work—I imagine if he had to be trying to do what I’m doing for this part of it then it would feel overwhelming, so we divide and conquer. (P4)	Work schedule = less focused attention on exercises Daycare

## Data Availability

The original contributions presented in this study are included in the article. Further inquiries can be directed to the corresponding author.

## Supplementary Materials

The following supporting information can be downloaded at: https://www.mdpi.com/article/10.3390/children11060689/s1, Interview Template/Questions for Parent Participant.

## Abbreviations

CMT	Congenital muscular torticollis
HEP	Home exercise program
PT	Physical therapy or physical therapist
ROM	Range of motion
CPG	Clinical practice guideline
