# Supplementary material for: A Qualitative Investigation of Parent Perceptions of Home Exercises for Congenital Muscular Torticollis
Source: Children (Basel). 2024 Jun 5;11(6):689. doi: 10.3390/children11060689 (PMC11201539; doi:10.3390/children11060689)
Supplement: Supplementary file 1 [file children-11-00689-s001.zip › children-3029591-supplementary.pdf]

Interview Template/Questions for Parent Participant

- Thank you for agreeing to speak with me about your baby's home therapy exercises for CMT.
- If you feel that you need a break at any time, we can stop the audio-recording and return to it at a later time.
- The interview will take about 15 minutes and includes 3 sections for this first interview.
- There will be 2 more interviews of about 10-15 min over the next 2 months.
- Do you have any questions about the study before we begin?

Section 1 -- 1st interview only.

I have some background questions for you:

a. What is (Baby's) current age?
b. At what age did (Baby) get a diagnosis of CMT?
c. At what age did (Baby) begin PT?
d. How often is (Baby) getting PT?
e. Is this your first child?

Section 2 (15 min): **<u>Interview template</u>**

We are going to talk about each of the (3) exercises/activities that you were prescribed by your physical therapist this month. "tummy time", "head turning", and "ear to shoulder"….are those the ones you are doing?

**Exercise Name &/or Activity Description**	**Approximate length of time completed each time (minutes)**	**# days completed each week**	**Comments:**<br>• **Thoughts**<br>• **Feelings**<br>• **Barriers / difficulties / stress**<br>• **Hand placement?**<br>• **I did not do this one because…or I did this one more because…**<br>• **Did your partner assist with these?**<br>○ **If offers info – "tell me more about that"**
Tummy time			
Ipsi-lateral flexion			
Contralateral rotation			
Other	• Did you feel like you were a part of the decision making process as it relates to these exercises? How so?<br>• Do you feel that you are handling your baby in a way that's comfortable for her/him.<br>• Do you feel confident in your ability to help baby move into different positions comfortably?<br>• Can you tell when baby needs a break? How? (3 bullets —Palmer et al, 2019)<br>• How are the baby's feedings going?<br>• How many times a day (an hour) does baby spit up? (Greve et al, 2022)<br>• Is baby active? (Kahraman et al, 2021)<br>• Have you felt a lump on baby's neck?<br>• How much help do you have at home?<br>• What would you want new parents to know about this condition? What does it feel like to have a baby with this health condition? Any a-ha moments you'd like to share?<br>• Thank you for your time. A member of the research team will contact you next month.		
